# Bone Apatite Nanocrystal: Crystalline Structure, Chemical Composition, and Architecture

**DOI:** 10.3390/biomimetics8010090

**Published:** 2023-02-22

**Authors:** Bin Wang, Zuoqi Zhang, Haobo Pan

**Affiliations:** 1 Shenzhen Institute of Advanced Technology, Chinese Academy of Sciences, Shenzhen 518055, China; 2 Department of Mechanical Engineering, City University of Hong Kong, Hong Kong SAR, China; 3 School of Civil Engineering, Wuhan University, Wuhan 430072, China

**Keywords:** bone apatite nanocrystal, crystalline structure, chemical composition, geometry

## Abstract

The biological and mechanical functions of bone rely critically on the inorganic constituent, which can be termed as bone apatite nanocrystal. It features a hydroxylapatite-like crystalline structure, complex chemical compositions (e.g., carbonate-containing and calcium- and hydroxyl-deficient), and fine geometries and properties. The long research with vast literature across broad spectra of disciplines and fields from chemistry, crystallography, and mineralogy, to biology, medical sciences, materials sciences, mechanics, and engineering has produced a wealth of knowledge on the bone apatite nanocrystal. This has generated significant impacts on bioengineering and industrial engineering, e.g., in developing new biomaterials with superior osteo-inductivities and in inspiring novel strong and tough composites, respectively. Meanwhile, confusing and inconsistent understandings on the bone mineral constituent should be addressed to facilitate further multidisciplinary progress. In this review, we present a mineralogical account of the bone-related ideal apatite mineral and then a brief historical overview of bone mineral research. These pave the road to understanding the bone apatite nanocrystal via a material approach encompassing crystalline structure, diverse chemical formulae, and interesting architecture and properties, from which several intriguing research questions emerge for further explorations. Through providing the classical and latest findings with decent clearness and adequate breadth, this review endeavors to promote research advances in a variety of related science and engineering fields.

## 1. Introduction

The biological and mechanical functions of bone rely critically on the inorganic constituent, which belongs to calcium- and phosphorous-containing apatite minerals. Besides providing high stiffness for mechanical support and protection, the bone inorganic phase stores and regulates many life-essential elements, e.g., 80 wt% of total body phosphorus (considering that phosphorus is present in DNA, RNA, and many important proteins), 99 wt% of calcium, and 50 wt% of magnesium [1,2,3]. Extensive studies have been devoted to understanding the composition, structure, and formation of bone minerals for developing new biomaterials with improved biocompatibilities; for example, synthetic bone substitutes using carbonated hydroxylapatite [4,5] and carbonated apatite [6,7] mimic the bone mineral more accurately and result in larger amounts of bone formation than stoichiometric hydroxylapatite and β-tricalcium phosphate. Meanwhile, the inorganic constituent serving as the reinforcing phase of bone has attracted increasing attention [8,9,10], due to the ingenious structures largely associated with the inorganic reinforcement leading to superior mechanical performance. This has provided bioinspired designs for developing novel strong and tough composite materials [11,12,13] for various aviation/aerospace, marine, and automobile industries.

This bone inorganic constituent can be considered a hydroxylapatite-like, poor-crystalline, and non-stoichiometric single solid mineral belonging to the apatite series, up to current understanding with vast literature across various disciplines and fields such as chemistry, crystallography, geology and mineralogy, biology and medical sciences (e.g., orthopaedics and dentistry), and materials science [2,14,15,16,17,18,19,20,21,22,23,24,25,26,27,28,29,30,31,32,33,34,35,36,37]. It is carbonated (containing 2–9 wt% carbonate as a substituent [24,25,28,32,36,38,39,40,41,42]), has a varying Ca/P molar ratio (also termed as calcium-deficient), is hydroxyl-deficient, and contains HPO_4_^2−^, structural water, and other minor substituent ions such as Na^+^ and Mg^2+^. This inorganic constituent of bone has been confirmed to have a nanometer length scale [2,9,26,27,28,33,43]. Thus, it is termed as bone apatite nanocrystal throughout this review (the small bones of the inner ear, e.g., otoconia, are an exception, being composed of vaterite [34,44]). A detailed justification for such naming rather than others, including hydroxyapatite, dahllite, and carbonated hydroxyapatite, is provided in Supplementary Materials. The following contents also subtly explain this.

The bone apatite nanocrystal has been investigated much more intensely than crystals in other vertebrate mineralized tissues [2]. Nevertheless, there have long been controversies on the chemical composition, formula, and crystalline structure of bone inorganic constituent from mineralogy, crystallography, and structural biology fields, as well as complex and confusing usages in naming this substance in much broader fields. For examples, apatite, hydroxyapatite, carbonated apatite, carbonate apatite, dahllite, carbonated hydroxy(l)apatite, carbonate hydroxy(l)apatite, and biological apatite have been used to describe the bone inorganic constituent in general fields of materials science, bioengineering/biomedicine, and mechanics communities. Furthermore, the challenging nature of bone mineral research, e.g., the crystallinity, composition, and lattice parameters can vary within the same bone [45], also complicates the situation. The extensive studies scattered and inconsistent in different fields on the same topic may cause confusions and further hinder effective multidisciplinary research and innovation.

This work aims to present a consolidated understanding encompassing a mineralogical account of the ideal bone-related mineral, a historical overview of bone mineral research, and a material approach addressing the bone apatite nanocrystal. We endeavor to provide the classical and latest findings with decent clarity and adequate breadth on this subject, so as to promote further research advances in a variety of bone mineral-related fields and disciplines.

## 2. Bone-Related, Ideal Hydroxylapatite: A Mineralogical Account

A brief, clear account of the ideal crystalline apatite and hydroxylapatite from a mineralogical perspective is indispensable to understanding the chemical composition and crystalline structure of bone apatite nanocrystals. Note that hydroxylapatite is used to conform to the majority of publications concerning the bone mineral across various relevant research fields and the suggestion on nomenclature for the apatite minerals from the International Mineralogical Association (IMA) [46,47] (hydroxylapatite with an "l" is the accepted term by the IMA [29]).

Hydroxylapatite is one specific apatite having an apatitic structure and well-defined chemical composition and formula. Apatite is a general mineral name representing a supergroup of minerals (more than forty species) that share one common crystalline structure but have different elements. Within this apatite supergroup there is apatite group which consists of about sixteen species of minerals. Within the apatite group there is apatite subgroup that denotes specifically three mostly known minerals, fluorapatite (FAp), hydroxylapatite (OHAp), and chlorapatite (ClAp) [25,26,29,30,34,35,47,48]. Historically, apatite was first introduced by Werner [49] in the mineralogical literature, and since 1856–1860, the three classical species of apatite of FAp, OHAp, and ClAp have been widely known [47]. The nomenclature of apatite updates as the number of mineral species with the same apatitic structure increases over the years. Therefore, in current mineralogy, apatites in a broad sense refer to the supergroup of minerals showing the same apatitic structure/atomic arrangement but with diverse chemical compositions, exhibited by a general chemical formula of M_10_(TO_4_)_6_X_2_. In this formula, M could be Ca^2+^, Ba^2+^, Pb^2+^, Sr^2+^, Mn^2+^, etc., T could be P^5+^, As^5+^, V^5+^, Si^4+^, and X could be (OH)^−^, Cl^−^, F^−^, O^2−^, etc. While apatites in a narrow sense specify the three calcium phosphate minerals, fluorapatite (Ca_10_(PO_4_)_6_F_2_), chlorapatite (Ca_10_(PO_4_)_6_Cl_2_), and hydroxylapatite (Ca_10_(PO_4_)_6_(OH)_2_), which have relatively determined chemical compositions.

Apatites are known for having a remarkably high tolerance of foreign elements and vacancies and a robust crystalline structure, e.g., more than half of the total number of elements in the periodic table can be substituted into the apatite crystal structure [50]. This further diversifies the number of possible real chemical compositions of apatites, since the general formula already includes a wide range of specific elements. The hydroxylapatite is no exception in this significant substituent-accommodating feature, tolerating various single- and multi-element cationic and anionic ions. This has been correlated to the biological functions of the bone mineral such as solubility and bone remodeling [3,29,51]. The crystalline structure of pure hydroxylapatite is described below due to its most similarity to that of the bone apatite nanocrystal.

**Figure 1:**
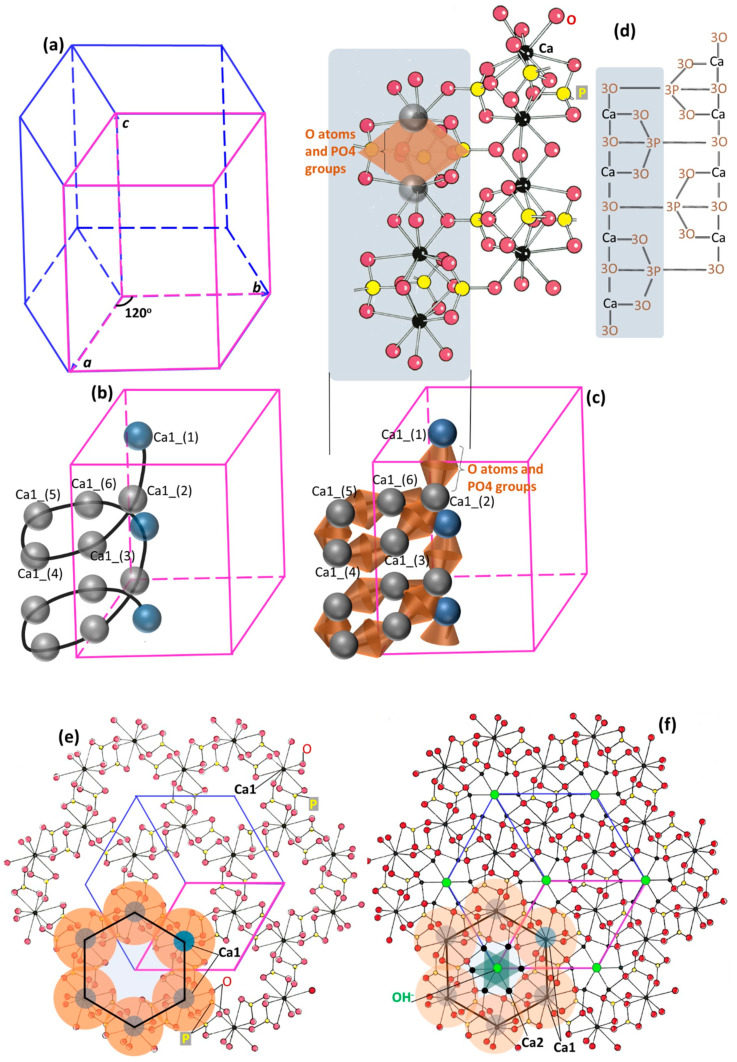
A step-by-step illustration of the crystalline structure of hydroxylapatite. (**a**) A hexagonal lattice, with the pink-colored frame representing the unit cell. (**b**) The Ca1 chains form 6-fold screw axis symmetry along each column; here only shows one Ca1 chain along one column. (**c**) Each Ca1 chain is a Ca-O-P chain, with the Ca atoms connected by O atoms and PO_4_ groups. (**d**) Each Ca1 chain is a Ca-O-P chain, with the Ca atoms connected by O atoms and PO_4_ groups above and below. The same PO_4_ groups link neighboring Ca1 chains. (**e**) Projection down the *c*-axis, showing the hexagon formed by the Ca1 chain with Ca atoms as nodes. The O atoms and PO_4_ groups are represented by orange-colored regions. Neighboring Ca1 chains link by the same PO_4_ groups and share two Ca1 atoms to form robust structure. (**f**) Projection down the *c*-axis, showing hydroxylapatite structure: Ca2 atoms fill into the helical Ca1 chains around the column and OH^−^ anions reside in the columns. (**d**–**f**) are adapted from [56].

Hydroxylapatite is predominantly hexagonal (the other type being monoclinic [52]), and has a space group symmetry of *P*6_3_/m (*P*6 means primitive hexagonal, 6 means order of the screw axis/rotation by an angle of 360°/6 = 60°, 6_3_ means translation of 3/6 = 1/2 of the unit cell length parallel to the *c*-axis, and m means the mirror plane perpendicular to the *c*-axis). The lattice parameters are *a* = *b* = 9.432 Å, *c* = 6.881 Å, and *γ* = 120° [53,54,55] (Figure 1a). The unit cell, which is the smallest representative unit of the general structure (pink-colored profiles in Figure 1a–d), contains elements that make up a complete formula of Ca_10_(PO_4_)_6_(OH)_2_. This formula format and stoichiometry correlate with the atomic arrangement in the hydroxylapatite structure. Within a unit cell, there are three types of crystallographic positions (corresponding to different roles in constructing the hexagonal structure): the calcium (Ca) sites which consist of Ca1 and Ca2 types, the tetrahedral sites (*T* sites) occupied by the PO_4_ groups, and the channel sites where (OH)^−^ anions reside.

The crystalline structure of hydroxylapatite is illustrated in a step-wise manner with increasing complexity in spatial arrangement and element types here (hoping to be comprehensible to wider research communities), based on previous crystallographic accounts [29,34,47,48,56]. This hexagonal structure is the fundamental reason for both structural robustness and high ion tolerance to fulfill many demanding biological and mechanical functions. Figure 1a shows a simple hexagonal system for hydroxylapatite. The vertical columns parallel to the *c*-axis direction are 6_3_ screw axes (meaning that rotation by an angle of 360°/6 = 60° and translation of 3/6 = 1/2 of the unit cell length parallel to the *c*-axis obtain the original motif). The Ca (in Ca1 sites) and oxygen (O) atoms and PO_4_ groups form Ca1 chains that screw-rotate around the columns (Figure 1b,c, only one Ca1 chain is shown). The detailed Ca1 chain structure, Ca atoms connected by O atoms and PO_4_ groups, is illustrated in Figure 1c,d. Figure 1d also shows the linking between neighboring Ca1 chains by the same PO_4_ groups. When projected down the *c*-axis, each Ca1 chain forms a hexagon with Ca atoms as nodes, shown in Figure 1e, with the O atoms and PO_4_ groups indicated by orange-colored regions. Such hexagonal shapes on the *a*-*b* plane actually form hexagonal columns in space, which have column walls and form channels along the *c*-axis direction. Figure 1e also reveals that the linking and the structure-forming between neighboring Ca1 chains are the PO_4_ groups and the sharing of two Ca1 atoms. Thus, the Ca1 chains and the PO_4_ groups construct the hexagonal skeletal framework and account for the great stability of the apatite structure [24,57]. Then, the Ca2 atoms fill into the column walls and two (OH)^−^ anions reside at the column central axis (at 0,0,1/4 and 0,0,3/4, with certain distortions [55], the so-called anion channel) in such a manner that the Ca2 atoms are arranged in two triangles (semi-transparent dark green colored profiles in Figure 1f) surrounding the two (OH)^−^ ions. These Ca2 and channel sites are different from the hexagonal framework-forming Ca1 and *T* sites. Vacancies can readily occur in the channel sites [58] and the Ca-deficiency is predominantly in the Ca2 sites [28,59,60,61]. However, vacancies in the *T* sites have never been reported [33], which was theoretically demonstrated to strongly destabilize the structure [62]; while substitution in Ca1 sites may lead to a lowering of the space-group symmetry (changing crystalline structure) [26] and the Ca1 occupancy is preserved when other ion substitutions take place (enamel as one complex carbonated bioapatite) [28,61]. All of these together form the full hydroxylapatite structure (Figure 1f, projection down the *c*-axis), giving rise to the structural robustness and compositional variousness. Figure 1d–f are adapted from [56].

## 3. A Brief Historical Overview of Bone Mineral Research

For a concise research overview, since the 18th century, our understanding of the bone inorganic constituent has evolved from Calcium phosphates **→** Apatite-like crystal **→** Hydroxylapatite-like structure → Diverse in-depth studies on (i) effects of carbonate substitution, varying Ca/P ratio, hydroxyl deficiency, and inclusions of other ions and molecules; (ii) chemical formulae; (iii) other possible mineral phases; (iv) shape, size, alignment of bone apatite crystals; → Core-shell structure of bone apatite nanocrystals, etc. For detailed and comprehensive historical overviews of research on bone inorganic phase, please refer to remarkable works such as [32,35,63,64,65,66].

The bone inorganic compound has been studied for more than 250 years, since recognizing the calcium phosphates in bone dates back as far as to 1769 (the Swedish chemists, Johan Gottlieb Gahn and CarlWilhelm Scheele, obtained tricalcium phosphate from burned bone and extracted the element of phosphorus from treated bone) [66,67]. Most of studies on bone minerals in the mid-1890s tried to analyze the elemental compositions by chemists, primarily using bone ash and then using pulverized bone [15,68]. During this time, the existence of carbonate and structural water in bone mineral were found, in addition to the established knowledge of dominant calcium phosphates [14] and other suspected phases such as amorphous calcium phosphate [69], octacalcium phosphate (OCP), and dicalcium phosphate dihydrate (DCPD) [32,65,70].

Then a milestone in bone mineral research was identifying it to be apatite-like crystal structure through X-ray diffraction firstly by de Jong in 1926 [17] (the apatite was known as a phosphate mineral [71]). This was made possible by the discovery and application of X-ray radiation in the early twentieth century [72,73], which shifted bone research from descriptive elemental composition to structural chemistry by crystallographers and mineralogists in the first half of the twentieth century [32,65]. The crystalline apatitic structure of bone mineral was verified later (very similar to diffraction patterns of fluorapatite, a model apatite) with successive findings [18,19,20,66,74,75,76], e.g., Taylor and Sheard reported the bone apatite to have very small crystal sizes [18], and Roseberry et al. found it structurally similar to dahlite (carbonated apatite) [20]. With the crystalline structural details (i.e., lattice parameters) of fluorapatite, chlorapatite, and hydroxylapatite [53,54,77] determined during this time, the bone mineral constituent was considered to mostly approximate hydroxylapatite (be a hydroxylapatite-like substance) [21,74,78], while other proposals, such as hydrated tricalcium phosphate compound [79,80], did not prevail.

Ever since the 1940s, it has been generally accepted that the bone mineral constituent most probably resembles the crystalline hydroxylapatite containing an appreciable amount of carbonate [21,22,25,26,56,57,81,82,83]. This has been widely used in current literature in disciplines of medicine, biology, physics, and chemistry, spanning a wide spectrum of related research fields. The important differences, such as chemical compositions, lattice parameters/distortions, crystal size and shape, and solubility, between pure hydroxylapatite (having a fixed structure and composition) and bone mineral phase are carefully kept in mind by researchers primarily in mineralogy and geology, and some in medical sciences, as demonstrated by the rigorous description “the bone mineral substance is apatite-like closely related to/resembling hydroxylapatite” e.g., [2,19,22,24,25,26,28,29,30,31,32,34,35,48,84,85,86]. While in biomaterials, biomedical sciences, materials and mechanics, histology, and anatomy, the bone inorganic constituent is often referred to as crystalline hydroxylapatite (overlooking the above-mentioned essential differences) or confused due to different names used [37]. This might be partially due to (i) the success of using hydroxylapatite as bone implant and grafting materials in terms of facile fabrication and good tissue response and osteoconductivity since the 1970s [30,35,65,87,88,89,90] and (ii) limited effective communications across the fields. Descriptions bearing the bone mineral features that affect the biological and mechanical properties of bone (e.g., bone apatite nanocrystal in this review) will show up as more interdisciplinary publications on this subject appear over time.

Since the 1950s, there have been numerous studies on the bone mineral substance, the carbonate substitution, the varying Ca/P ratio, the hydroxyl deficiency, the inclusion of structural water and other minor ions such as HPO_4_^2-^ in various fields of chemistry, minerology, geology, medical sciences, and materials, using more advanced characterization techniques (e.g., X-ray with Rietveld refinements, Raman spectroscopy, Fourier Transform-Infrared spectroscopy, Laser Raman spectroscopy, Nuclear Magnetic Resonance and its variants) [24,25,26,28,29,31,32,35,48,86]. The effects of the above features on the lattice parameters [25,83,91], the crystallite size and shapes [36,83], and other chemical and physical properties such as stability [29,85], solubility [92], and elastic mechanics [93,94,95,96,97] have been examined using not only bone apatite nanocrystals but also geological and synthetic apatites to better control the experimental conditions to explore the mechanisms.

## 4. Bone Apatite Nanocrystal: A Material-Approach Account

### 4.1. Crystalline Structure

**Figure 2:**
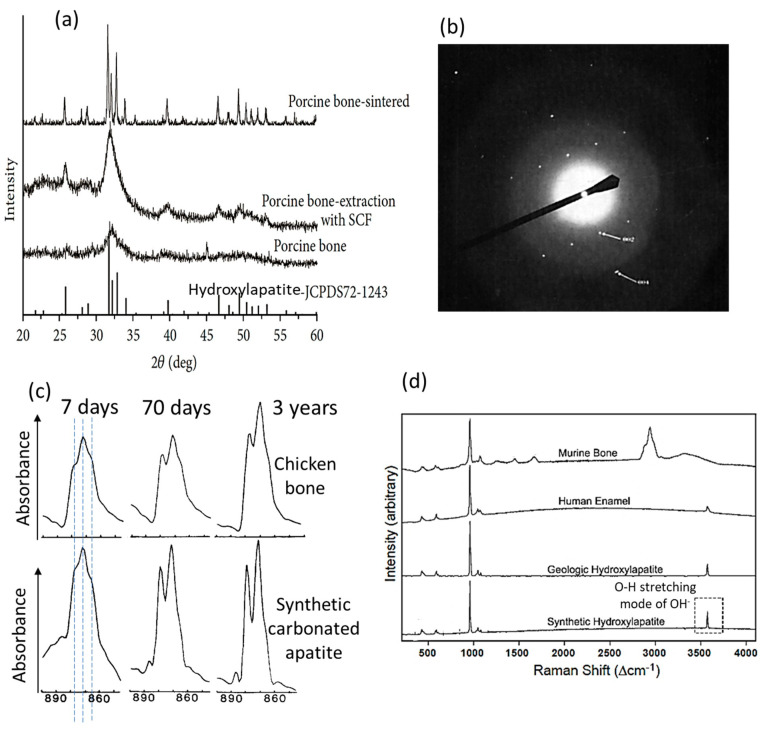
Structure and composition of the bone apatite nanocrystal. (**a**) X-ray diffraction patterns of porcine bone with different treatments in comparison with the standard PDF card of hydroxylapatite [99]. (**b**) Electron diffraction pattern of single isolated inorganic crystals from bovine bone, showing reflections typical of apatite [101]. (**c**) Resolution-enhanced FTIR spectra of chicken bone (upper row) and synthetic carbonated apatite (lower row), showing the increasing intensities of CO_3_^2−^ in the $\nu_{2}$ domain with increasing time (at 7 days, 70 days, and 3 years) [107]. (**d**) Raman spectra of a cortical mouse bone, the enamel of a human molar, geologic apatite, and synthetic hydroxylapatite [29]. The spectra of enamel, synthetic, and geological hydroxylapatite show clear O-H stretching models around 3572 cm^−1^ for hydroxyl (OH^−^), but those of bone apatite do not.

The address on the apatite and hydroxylapatite crystalline structure and a historical overview of bone mineral research pave the road to understanding the numerous, specific studies on bone apatite nanocrystals. Firstly, the bone inorganic compound has long been identified to have an apatite structure (with a hexagonal unit cell since de Jong [17,26,28,98]) based on X-ray diffraction patterns most similar to those of crystalline fluorapatite and hydroxylapatite but with much broadening of the peaks (Figure 2a [99]). This corresponds to the hydroxylapatite-like and poor-crystalline nature of bone apatite nanocrystals. Such X-ray results indicate the low crystallinity, the lattice distortions and imperfections caused by many foreign ion and molecule substitutions, and the extremely small size (e.g., several nanometers/only a few atomic layers) of the bone apatite crystals [24,26,100]. This, plus the difficult elimination of interference from intimately bonded organic macromolecules without introducing additional artifacts [101], makes bone inorganic constituent extremely difficult to study. Despite the broad diffraction patterns of bone that restrain precise determination of lattice parameters, there are reported measurements of the *a*-axis dimension ~9.419 Å ± 0.001 Å [102], which were correlated to the substituent ions [103]. In addition, it is found that the X-ray diffraction peaks of bone are in more resemblance to those of poorly crystalline hydroxylapatite synthesized at room temperature [100]. Additionally, changing the crystallinity and crystal size through different treatments, e.g., extraction with supercritical fluid CO_2_ (to dissolve the organic components) and sintering (to allow crystal growth), have led to increased sharpness of the diffraction peaks, which approaches those of the crystalline hydroxylapatite [99] (Figure 2a). Meanwhile, aging of bone increases the crystallinity and crystal size [31,39,104,105]. These support the widely held belief that the majority of the bone mineral substance is most probably hydroxylapatite. The electron diffraction pattern of isolated, single crystals from bovine bone shows typical apatite reflections (shown in Figure 2b [101]), corroborating the apatitic structure determined from X-ray diffractions. It is also noted that the monoclinic hydroxylapatite might occur in biological apatites such as the mineral substances in bone and teeth, as a loss of hexagonal structure was reported [106], but this needs more investigation [28].

### 4.2. Chemical Composition

The bone apatite nanocrystal shows a wide range of chemical compositions due to remarkably tolerating various ions and vacancies (allowed by the apatitic structure) and adsorbing foreign ions and molecules (by the nanometer size with a large surface/volume ratio). This corresponds to the non-stoichiometric nature of bone apatite. It has been well-known that bone apatite has a significant amount of carbonate, 2–9 wt%, firstly identified as substitutive ions by McConnell [38] and also by many researchers such as [25,28,29,41,42,48,108,109,110]. Thus, bone apatite has been termed variously as carbonate apatite [87,111,112], carbonated apatite [113], or carbonated hydroxyapatite [37,114], and calcium-deficient apatite [100]. Dahllite was used to denote carbonate-bearing apatite (for carbonate/carbonated apatite and carbonated hydroxyapatite) following the geology community, but currently, the IMA does not recognize dahllite as a distinct mineral since the CO_3_^2−^ ions are not known as the dominant anion species [29]; while calcium-deficient apatite overlooks other important features of bone apatite crystals such as carbonate substitution and hydroxyl deficiency. With detailed justifications in Supplementary Materials, bone apatite nanocrystal is assigned to the solid inorganic substance of bone.

In bone apatite crystal, the carbonate ions could substitute for the OH^−^ sites (A-type substitution), for the PO_4_ sites (B-type substitution), or for both, among which the number of B-type substitution prevails over that of A-type substitution [26,28,115,116,117]. Studies also show that the A-type substitution could be favored under certain conditions [116]. The common substitutions observed in the bone mineral are the coupled substitutions of CO_3_^2−^ for PO_4_^3−^ and Na^+^ for Ca^2+^ with vacancies on the calcium and hydroxyl sites [24,28,42] to maintain charge neutrality. The degree of carbonate substitutions in bone apatite increases with aging; as shown in Fourier Transform Infrared (FTIR) spectra of bone in Figure 2c [107], the intensity and sharpness of the absorption bands around 879 cm^−1^ representing CO_3_^2−^ (ν_2_ mode, strong [28] and free from organic interference [117]) increase with the age of the chicken bone and with maturation for the synthetic crystalline apatite. The band representing the B-type substitution (at 871 cm^−1^) shows a higher intensity than that representing the A-type substitution (at 878 cm^−1^), and carbonate ions that are not within stable/crystalline sites were also identified (shoulder at 866 cm^−1^) [107]. This incorporation of carbonate causes changes in crystalline apatite structure due to the differences in ion size, coordination, and bondings. For instance, replacing the PO_4_^3−^ with CO_3_^2−^ decreases the *a*-axial length of the unit cell [91] and the overall crystallite size [118,119,120], and increases the *c*-axial length [83] and the amount of crystallographic microstrain [29,92]; together with the fact that the Ca-CO_3_^2−^ bonding is weaker than the Ca-PO_4_^3−^ bonding [28], the carbonate substitution reduces the thermal stability and increases the solubility of carbonate-containing apatite [26,29,121]. The extent of these effects is also influenced by other ion substitutions [24,122], and such effects are considered favorable for bone remodeling, homeostasis, and other biological functions [85]. Recent studies also show that the carbonate substitution plays an important role in constraining the size and regulating the formation and shape of the bone-like apatite nanocrystals [36,123,124]. The carbonate substitution-induced changes in apatite lattice parameters and effects on related physiochemical properties such as solubility [83,91,125] have provided important fundamentals for this bone apatite morphology control. It has been frequently demonstrated that the carbonate-containing apatite materials and scaffolds produce superior bone maturation and formation properties than non-carbonate doped ones [5,6,126]. This has led to new commercial artificial bone materials (e.g., Cytrans^®^) [7] and carbonate apatite approved for clinical use [127].

Additionally, carbonate substitution affects the elastic properties of the bone apatite nanocrystals, e.g., increasing the carbonate content decreases the bulk modulus and elastic strain ratio revealed by experiments [94,97,128,129] and decreases the elastic constants/modulus from computational simulations [36,96,130,131,132]. These can provide important insights into modulating the mechanical function of bone and developing bone implant biomaterials and bone-inspired engineering composites.

The varying Ca/P molar ratio and associated vacancies and substitutions on the hydroxyl sites lead to Ca-deficient [29,35], hydroxyl-deficient, and HPO_4_^2−^ containing bone apatite nanocrystals. The varying Ca/P molar ratio could be caused by ion substitutions such as the removal of Ca^2+^ (Ca deficiency) to balance the charge when replacing PO_4_^3−^ by CO_3_^2−^ (dominate) or HPO_4_^2−^ ion adsorptions on the nanocrystal, and the possible presence of other phases with the bone apatite [24,57], although the co-existence of other phases has been debated frequently [28,31]. Studies on the incorporation of carbonate and HPO_4_^2−^groups [23,133] reveal ‘non-apatitic/labile environments’ (non-crystalline regions) of the CO_3_^2−^ and HPO_4_^2−^ groups, and the HPO_4_^2−^ groups in bone nanocrystals are different from those in synthetic carbonate- and HPO_4_^2−^ containing apatites [134]. The general HPO_4_^2−^ containing apatites can have a varying Ca/P molar ratio from 1.5 to 1.67 [29], as represented by a formula of Ca_10−x_[(PO_4_)_6−x_(HPO_4_)_x_](OH)_2−x_ (0 ≤ x ≤ 2) [135] (1.67 is for the crystalline hydroxylapatite), while these variations do not result in apparent changes in the X-ray diffraction patterns [26]. It is worth noting that the above formula (usually from materials research fields) implies the Ca deficiency or the varying Ca/P ratio caused by the HPO_4_^2−^ substituting for PO_4_^3−^ (Ca/P ratio equal to (10 − x)/6) [35]; however, for bone apatite, the dominant substitution is carbonate, and a recent study found that the HPO_4_^2−^ ions are concentrated at the surface of the bone apatite mineral crystals rather than the inside [136]. Therefore, better formulae should consider both types of ion incorporations and their amounts and effects in changing the Ca/P ratio. Chemical analyses on synthetic bone-like apatites reveal that the Ca vacancy occurs predominantly at Ca2 sites [28,59,60,61]. Notably, the carbonate substitution, HPO_4_^2−^ content, and Ca deficiency were found to be age-related, e.g., the CO_3_^2−^ content increases, the HPO_4_^2−^ decreases, and the Ca/P ratio increases (from 1.51 at birth to 1.69 in the adult, by a constant calcium content but decreasing phosphate ion content) with maturation [39]. It has also been recognized that the ‘age’ affecting the composition, structure, and interaction properties of bone apatite nanocrystals is principally the age of the crystals, the time elapsed after the initial crystal deposition within the organic matrix (till removal/resorption of crystal) [2,31].

The hydroxyl deficiency in bone apatite nanocrystals, first found over fifty years ago [137,138], has been quantified in recent decades, about 80–85% less in hydroxyl groups [139,140] or none at all [141] compared with crystalline hydroxylapatite. This hydroxyl deficiency [29,31,141] might be less recognized by most materials and biomechanics communities. As shown in Figure 2d, the murine bone apatite shows a Raman spectrum that is similar to that of human enamel, geologic hydroxyapatite, and synthetic hydroxyapatite, indicating structural similarity. However, the bone apatite does not show the stretching modes of hydroxyl at the position around 3570 cm^−1^ (dotted rectangle in Figure 2d). This is representative of all cortical bone from different mammals investigated by [29], and some bone apatite may not have any hydroxyl groups at all [29], which have been consistently found by Raman and infrared spectroscopies, inelastic neutron scattering, and nuclear magnetic resonance spectroscopies [84,117,139,141,142,143]. This hydroxyl deficiency distinguishes bone apatite from synthetic, crystalline hydroxylapatite. Results from proton-NMR and FTIR also show that the bone apatite crystals do not contain hydroxyl groups [141,144]. It is proposed from examining synthetic hydroxylapatites with varying hydroxyl deficiencies that smaller crystal size and higher atomic disorder generate apatites that are less favorable in taking hydroxyl ions into the channel sites (a higher hydroxyl-deficiency degree) [142]. This can further explain the different amounts of hydroxyl groups in bone, dentin, and enamel in correlation to their different crystallinities and crystal sizes. Additionally, the bone apatite contains about 3 wt% lattice-incorporated water molecules within the channel sites [85,145,146], in addition to the hydroxyl groups and different from the water molecules adsorbed on the bone apatite nanocrystal surface. Water as a component of the bone apatite had been found via chemical analysis since the 1870s, proposing more than 1 wt% H_2_O within the crystalline apatitic structure [15,63,68,147], and discussed ever since [32,57]. Although this structure-incorporated water might not have gained equal research attention as other ion substitutions such as the carbonate in the twentieth century, it has been re-recognized in recent decades [32]. There are also limited cationic substitutions such as Na^+^, Mg^2+^, and Sr^2+^ for the Ca sites, and anionic substitutions including F^−^ and Cl^−^ for the channel sites existing in the bone apatite nanocrystals [24,28,30,148]. F^−^ substitution for the OH^−^ does not change the atomic arrangements of hexagonal apatite but leads to a contraction in the *a*-axis dimension, while Cl^−^ substitution may result in changing the hexagonal to monoclinic symmetry [30].

With the complex chemical compositions and crystalline structure, many studies have been proposing models/chemical formulae for bone apatite nanocrystals (even before the twenty century), initially focusing on the role and function of carbonate and then incorporating the effects of Ca/P molar ratio, HPO_4_^2−^, hydroxyl deficiency, structural water, etc. Some of the notable chemical formulae are Ca_8_._3_(PO_4_)_4_._3_(CO_3_)_x_(HPO_4_)_y_(OH)_0_._3_, (x + y) equal to 1.7 [39], (Ca)_10−x_[(PO_4_)_6−x_(CO_3_)_x_](OH)_2−x_]∙1.5H_2_O [28,42,85,149] considering the effects of structural water, Ca_10−x_M_x_(PO_4_)_6−y_(HPO_4_)_y_(OH)_2_ [24,25,148], in which M represents cationic substitutions (e.g., Mg^2+^ and Na^+^) in the Ca sites, (Ca)_10−x_(PO_4_)_6−x_(HPO_4_,CO_3_)_x_](OH)_2−x_ (which shows that the vacancies at Ca^2+^ and OH^−^ compensate for the loss of negative charge by HPO_4_^2−^ or CO_3_^2−^ substituting for PO_4_^3−^) [33], and Ca_7_._5_(PO_4_)_2_._8_(HPO_4_)_2_._6_(CO_3_)_0_._6_ (OH)_0_._2_ [136].

There have been long debates on whether there are other inorganic phases, such as tricalcium phosphate (TCP, Ca_3_(PO_4_)_2_∙2H_2_O), amorphous calcium phosphates (ACP), dicalcium phosphate dihydrate (brushite, Ca(HPO_4_)∙2H_2_O), and octacalcium phosphate (OCP) or OCP-like solid phases [26,28,57,81,150,151,152,153,154], as transient/precursor phases or apatite-coexisting components within the bone mineral constituent. These could explain the broadening of bone X-ray diffraction patterns, the inconsistency between hexagonal structure and overall platelet shape, the presence of other ions such as HPO_4_^2−^, and the Ca-deficiency or varying Ca/P molar ratio. It is considered that strong circumstantial evidence exists for OCP participating in the nucleation of biological and thus bone apatite nanocrystals [26]. However, except in pathologic tissue calcifications [30], no direct evidence for OCP [27,31,84,155], crystalline brushite [28,156], or reproducible evidence for amorphous phases other than poor-crystalline apatite and its non-apatitic regions have been found [31]. This has been discussed in detail by Rey et al. [31] in terms of stressing that (i) direct, reproducible, and consistent results by different identifying methods for the existence of other phases are lacking, but consistent results for bone apatites abound; (ii) the initial state is poor-crystalline, unstable, super small bone apatite crystal (large surface area for adsorption, high capacity of ion substitution) that can undergo phase transitions when hydrated/disturbed; (iii) the existence of non-apatitic environments of bone apatite cannot be taken as the presence of other crystalline/amorphous precursor phases.

### 4.3. Geometry: Shape, Size, Architecture

**Figure 3:**
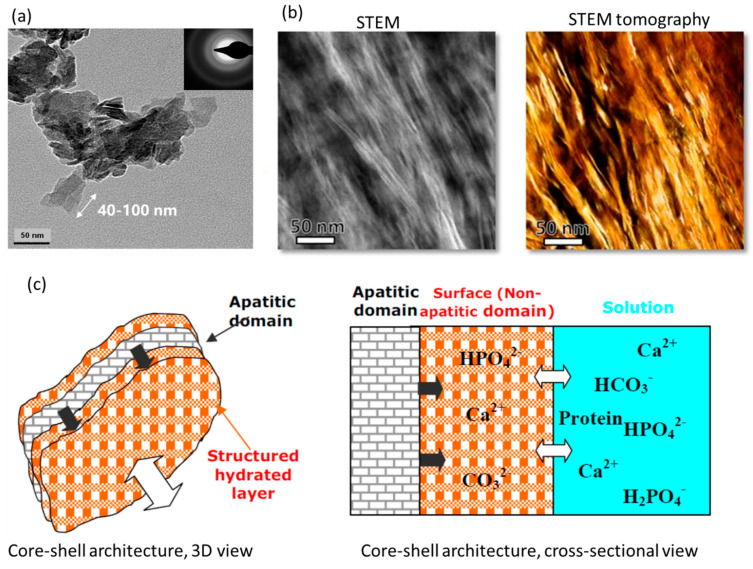
Geometry and architecture of the bone apatite nanocrystal. (**a**) TEM image of the inorganic crystal from bovine bone showing the nanoscale platelet shape [169]. (**b**) Scanning TEM image and STEM tomogram reconstructed from STEM images of bone apatite nanocrystals within the collagenous matrix, showing the nanoscale filamentous/acicular shapes [9]. (**c**) Schematic of the core-shell architecture of bone apatite nanocrystals [86].

It has been generally accepted that the bone apatite nanocrystal shows a plate shape elongated parallel to the *c*-axis of the hexagonal apatitic structure [82,157,158], despite controversies in the crystal morphology existing ever since the 1950s [33,101,159,160,161]. The plate shape has been reported, debated, and then verified by isolating the crystals of bone mineral constituent without introducing interfering damages/defects and imaging these directly using TEM and AFM [101,162]. Recent works have partially confirmed the plate shape and provide new complexities in morphologies of the bone apatite nanocrystal [9,43,114]. Earlier studies reported the needle/rod-shaped crystals of bone apatite [163,164,165], which were later demonstrated to be the edge profiles of the platelets by observing the bone apatite specimen with in-situ rotation [166,167,168]. Detailed historical overviews on this can be found in [2,28]. Figure 3a shows a TEM image [169] of the bone apatite crystals in the plate shape. Based on extensive reports, the plate-shaped bone apatite nanocrystals show dimensions of 15–100 nm in length, 10–45 nm in width, and 0.5~4 nm in thickness. The alignment, meaning the crystallographic *c*-axis vs. the longest dimension of bone apatite nanocrystal vs. collagen fibril axis, has been found to be parallel among each other [82,163,170,171,172]. While the recent work based on advanced 2D high-resolution transmission electron microscopies and 3D reconstruction [9] reports that the bone apatite nanocrystal shows multiple forms with different hierarchies: acicular (needle-shaped) crystals (5 nm and 50–100 nm long) (Figure 3b), platelets by partly merged acicular crystals (5 nm × 20–30 nm × 50–100 nm), and stacks of platelets (20–40 nm × 20–40 nm × 100 nm). These findings have been reasonably supported by a later study reporting the chiral structure of bone mineral including twisted needle-like crystals, subplatelets, and platelets [43], with similar length dimensions for each shape as those reported by [9].

One notable aspect is the explanation of the inconsistency between the hexagonal crystal structure and the non-hexagonal but platelet-shaped nanoscale morphology of the bone apatite crystal. This is very different from large-sized mineral crystals, which normally show consistent morphologies with their atomic crystal symmetry. For instance, geological hydroxylapatite exhibits large crystallites in hexagonal prisms derived from its hexagonal lattice structure [2,173]. Earlier studies hypothesized that the bone apatite crystals grow via a transitory precursor of octacalcium phosphate, which has a triclinic structure and shows plate-shaped crystals [81,113,150], although no direct evidence for the presence of octacalcium phosphate has been found (as discussed in the previous paragraph), even when directly examining the isolated bone apatite crystals [2,27,28,101]. From the compositional perspective, researchers found that [29,36,83] the carbonate substitution changes the lattice parameters and thus controls the nanoscale size and platelet morphology of bone apatite crystals, in addition to the prevailing hypothesis of organic-mineral interactions [174,175,176].

Concerning the nanometer scale of bone apatite crystal, there have been important explanations from different perspectives, in addition to (i) a general hypothesis that the nanocrystallinity may have a biological advantage and (ii) facilitating physiological functions through the large surface area for interfacial interactions and ion release [173,177]. From a mineralogical point of view [29,142], the appreciable CO_3_^2−^ substitution into the bone apatite crystal could constrain the size to be at the nanoscale with great atomic disorder generated inside [83,91]. This in turn could change the chemical composition by altering the crystallite’s ability to tolerate ions such as OH^−^, and further exert controls on the physical and biochemical properties (e.g., solubility). In materials and mechanics, such a nanometer size for the apatite crystal could be evolutionarily refined to ensure optimal strength and maximum flaw tolerance, as revealed through theoretical and numerical models [8,178,179,180]: the bone apatite nanocrystal mainly sustains tension while the organic matrix shears. This famous tension-shear-chain mechanical model and many other related works have constituted the important theoretical framework for the development of novel advanced composite materials. For examples, mimicking the structural features such as the geometry and alignment of bone apatite nanocrystals has led to many strong and tough bioinspired composites [11,13].

Interestingly, such a small nanoscale size allows the bone apatite to have an inherent architecture: amorphous, non-apatitic, surface layers sandwiching a crystalline, apatitic core (Figure 3c) [31,33,107,117]. This core-shell structure was primarily found by recognizing the ions having different local environments within the bone apatite nanocrystal, e.g., crystalline apatitic and amorphous non-apatitic, corresponding to the previously discussed incorporated HPO_4_^2−^ ions that do not have the same environments as those in other minerals. The surface layer of the bone apatite has been long found to be less crystalized compared with the inner core [33] due to the large surface effect from the super small size and the high affinity of adsorbing a variety of ions, molecules such as water, etc. The surface layers are reported to be in the form of hydrated amorphous calcium phosphate where HPO_4_^2−^ ions concentrate. By localizing the different ions and quantifying their contents within the bone apatite nanocrystal, a structural model with chemical details could be determined. The significant volume fraction of the amorphous surface layers, ~40%, may stimulate new thoughts in areas such as the biomineral-biopolymer interactions for biomineralization and bone implant development. The sizes of this interior sandwich structure have been estimated based on the location of different ions in apatitic (core) and non-apatitic (shell) positions, with approximately 0.8 nm-thick shells sandwiching a 2.4 nm-thick core [136]. Such hydrated surface layers also complicate the determination of a definitive formula for the bone apatite nanocrystal due to uncertainties about the compositions of the non-apatitic and apatitic parts [86], shown in Figure 3c.

### 4.4. Mechanical Properties

Studies on mechanical properties of the bone apatite nanocrystals have been much less than those on the compositional and structural research, probably due to the super small sizes restraining mechanical tests and the limited knowledge for analysis. Earlier studies usually used the elastic properties of geological hydroxylapatite [93,181] to estimate those of the bone apatite. Then, there have been computational [36,96,182] and experimental [94,97] studies investigating the elastic properties of bone apatites. Up to current understanding, the bone apatites are reported to have an elastic modulus of about 60–127 GPa (depending on the CO_3_^2−^ content from ~18 wt% to 0%) from simulations and experiments on synthetic bone-like carbonated hydroxylapatite [36,96,97], which are significantly higher than the experimentally measured values of 52–64 GPa from extracted fish bone mineral substances [94]. Even synthetic carbonated hydroxylapatite with a carbonate content as high as 17.8 wt% generates elastic modulus values (74–60 GPa) that are higher than the highest elastic modulus (63.9 GPa) of bone mineral substances normally with no more than 8 wt% carbonate content. This indicates that the mechanical properties of bone apatite are influenced not only by the carbonate substitution but also by other factors that need to be investigated in the future.

## 5. Conclusions and Outlook

The literature on the bone apatite nanocrystal has been immense, spreading into different disciplines and various research fields, e.g., mineralogy (including geology, earth sciences, crystallography, physics), biology and medicine (including histology, dentistry, orthopaedics, biomedical engineering, tissue engineering, biomedicine, drug delivery, osteoporosis, etc.), and materials and mechanics (including zoology, biological materials, biomaterials, biomechanics, mechanical modeling, bioinspiration, etc.). More questions, however, about the exact chemistry, crystallographic structure, and architecture of bone apatite nanocrystals remain and emerge, which are important for advances in a broader spectrum of fields aforementioned. Importantly, to leverage such a wealth of knowledge scattered in respective areas, the confusing (or even misleading) usages and inconsistent understandings of the bone mineral should be addressed to promote further multidisciplinary progress. For example, the general materials science and biomedical communities using hydroxylapatite for bone mineral constituent should be aware of the important features including carbonate-containing, poor-crystalline, non-stoichiometric with varying Ca/P ratios, and hydroxyl-deficiency, which could affect the efficacy/success of the related research activities.

Pertaining to the contents presented, there are several interesting aspects for further research. From biological and mineralogical perspectives, the bone apatite nanocrystal is both highly robust and remarkably tailorable, maintaining its stable crystalline structure while being able to dissolve to release essential elements and bond organic molecules; how to achieve these seemingly mutual exclusive features is very intriguing, which can provide new insights in developing smart materials for biomedical and robotics applications. In particular, apatite nanocrystals doped with rare earth elements, leveraging both the high structural stability and the wide chemical diversity, show unique luminescent and biological properties for various promising applications such as imaging probe/marker, bone tissue repair/regeneration, drug delivery, and antibacterial fields. In addition, studies have shown that biological and physical properties, including solubility, could be regulated by non-biological mechanisms such as ion substitution and crystal size management; this may stimulate new pathways for engineering better synthetic biomaterials with superior biocompatibilities free of complex cellular processes. For example, incorporating carefully-designed hydroxylapatite nanocrystals into the ink material for three-dimensional (3D) printing could produce bone tissue engineering scaffolds with better mechanical and biological/regenerative properties. This could be mostly due to an integration of capturing the important atomic and nanoscale features of bone mineral, such as carbonate and ion substitutions and shape control, and ensuring the micro- to mesoscale structural organization of bone from the mineral and organic components through 3D printing. Furthermore, the core-shell architecture of the bone apatite nanocrystal, amorphous layers sandwiching an apatitic core, also raises questions, e.g., how does the bone mineral with soft amorphous surface layers provide as high stiffness as previously assumed to be entirely crystalline apatite? This may offer important structure design strategies, e.g., stiffening and strengthening through geometry and/or structure rather than altering the material chemistry, for developing high-performance composite materials underpinning various engineering industries. Collectively, the contents and discussions in this review are only part of the vast, interesting, and important aspects of bone apatite nanocrystal research, which await more to be explored and exploited in the future.

## Data Availability

Data sharing is not applicable to this review article.

## Supplementary Materials

The following supporting information can be downloaded at: https://www.mdpi.com/article/10.3390/biomimetics8010090/s1, File S1: Naming the bone inorganic constituent to be bone apatite nanocrystal. References [183,184] are cited in Supplementary Materials File S1.
