# Supplementary material for: Bone Apatite Nanocrystal: Crystalline Structure, Chemical Composition, and Architecture
Source: Biomimetics (Basel). 2023 Feb 22;8(1):90. doi: 10.3390/biomimetics8010090 (PMC10046636; doi:10.3390/biomimetics8010090)
Supplement: Supplementary file 1 [file biomimetics-08-00090-s001.zip › biomimetics-2124849-supplementary.pdf]

Naming the bone inorganic constituent to be bone apatite nanocrystal, rather than choosing one of the various descriptions in literature:

(1) carbonate apatite, carbonated apatite, carbonated hydroxylapatite may raise confusion/questions about the composition, since they are not clear in indicating the role of carbonate inclusion, whether it is substituent or just compositionally involved [1-3]; the abbreviation of carbonate apatite, CO_3Ap [4], compared with that of hydroxylapatite, OHAp, may have misleading implications that the OH^- groups in the apatite structure are all replaced by carbonate.

(2) hydroxylapatite may have misleading implications that the bone inorganic constituent is a well-defined substance as hydroxylapatite; this is not true, because: (i) questions remain about both the exact chemistry and the exact crystallographic structure of bone mineral [1], and (ii) bone mineral differs from pure, crystalline hydroxylapatite in composition, in lattice parameters, in crystal size, in shape (synthetic, geologic hydroxylapatite show hexagonal prisms/rods, while bone apatite nanocrystals show nanoscale plate shapes), in solubility and dissolution (process) properties.

(3) bone hydroxylapatite is not appropriate because the bone inorganic phase, although showing X-ray characteristic to that of hydroxylapatite, exhibits few to no OH^- groups evidenced by many analytical techniques such as Raman and infrared spectra, inelastic neutron scattering, and nuclear magnetic resonance [5-9].

(4) biological apatite (bioapatite) is not appropriate because biological apatite is a general name including the apatite-like minerals in bone, dentin, enamel, and other biological calcified tissues. The apatite-like minerals in these biological calcified tissues are different in terms of composition, lattice parameters/crystallinity, crystal size and shape, and solubility.

(5) dahlite/dahllite was not chosen because currently the International Mineralogical Association does not recognize dahllite and francolite as distinct mineral names for carbonate apatite and carbonated fluorapatite (although dahlite and francolite were initially used by the geologic community to refer to carbonate-bearing hydroxylapatite and carbonate-bearing fluorapatite and these names continue to be used) [1].

The denotation of bone apatite expresses more features of the bone mineral phase belonging to the apatite (apatitic lattice structure, non-fixed chemical composition, carbonate substitution, Ca and OH deficiency, etc.) and meanwhile imposes specific apatitic features to bone by using "bone apatite nanocrystal".

References**:**

[1] Wopenka, B. and Pasteris, J.D., 2005. A mineralogical perspective on the apatite in bone. Materials Science and Engineering: C, 25(2), pp.131-143.
[2] D. McConnell, J.W. Gruner, Am. Mineral. 25 (1940) 157.
[3] D. McConnell, Am. J. Sci. 238 (1938) 296.

[4] Elliott, J.C. (1994): Structure and chemistry of the apatites and other calcium rthophosphates. Elsevier, Amsterdam,
[5] Pasteris, J.D., Wopenka, B., Freeman, J.J., Rogers, K., Valsami-Jones, E., Van der Houwen, J.A. and Silva, M.J., 2004. Lack of OH in nanocrystalline apatite as a function of degree of atomic order: implications for bone and biomaterials. Biomaterials, 25(2), pp.229-238.
[6] Rey, C., Collins, B., Goehl, T., Dickson, I.R. and Glimcher, M.J., 1989. The carbonate environment in bone mineral: a resolution-enhanced Fourier transform infrared spectroscopy study. Calcified tissue international, 45(3), pp.157-164.
[7] Rey, C., Miquel, J.L., Facchini, L., Legrand, A.P. and Glimcher, M.J., 1995b. Hydroxyl groups in bone mineral. Bone, 16(5), pp.583-586.
[8] Glimcher MJ (1998) The nature of the mineral phase in bone: biological and clinical implications. In Metabolic Bone Disease. Avioli LV, Krane SM (eds) Academic Press, New York, p 23-50.
[9] Loong, C.K., Rey, C., Kuhn, L.T., Combes, C., Wu, Y., Chen, S.H. and Glimcher, M.J., 2000. Evidence of hydroxyl-ion deficiency in bone apatites: an inelastic neutron-scattering study. Bone, 26(6), pp.599-602.
